# Actualized inventory of medicinal plants used in traditional medicine in Oaxaca, Mexico

**DOI:** 10.1186/s13002-020-00431-y

**Published:** 2021-01-28

**Authors:** Cruz-Pérez Alejandra Lucía, Barrera-Ramos Jacqueline, Bernal-Ramírez Luis Alberto, Bravo-Avilez David, Rendón-Aguilar Beatriz

**Affiliations:** 1 Universidad Autónoma Metropolitana Iztapalapa, Avenida San Rafael Atlixco 186, Colonia Vicentina, CP. 09340 CDMX, Mexico; 2 Universidad Autónoma de Querétaro, Avenida de las Ciencias, S/N, Juriquilla. Delegación Santa Rosa Jáuregui, CP 7623 Querétaro, Mexico

**Keywords:** Medicinal plants, Ethnic groups, Oaxaca, Diseases, Biodiversity, Cosmovision, Risk category, Gathering

## Abstract

**Background:**

Oaxaca is one of the most diverse states in Mexico from biological and cultural points of view. Different ethnic groups living there maintain deep and ancestral traditional knowledge of medicinal plants as well as traditional practices and beliefs about diseases/illnesses and cures. Previous ethnobotanical research in this state has helped document this knowledge, but with the addition of more studies, more records appear. We updated the inventory of medicinal knowledge between the different ethnic groups that inhabit the Oaxacan territory.

**Methods:**

A database was constructed from two sources: (1) original data from a 3-year project in 84 municipalities of Oaxaca inhabited by eight ethnic groups and (2) different electronic databases.

**Results:**

Records of 1032 medicinal plants were obtained; 164 families were registered, with Asteraceae, Fabaceae, and Rubiaceae being the most commonly used. A total of 770 species were reported in 14 vegetation types; the most important species came from temperate forests. Only 144 species corresponded to introduced species, and 272 were listed in a risk category.

Illnesses of the digestive and genitourinary systems as well as culture-bound syndromes were treated with high numbers of medicinal plants. The Mestizo, Mixe, Mixtec, and Zapotec ethnic groups exhibited the greatest number of recorded medicinal plants. The 17 species that were used among almost all ethnic groups in Oaxaca were also used to cure the highest number of diseases.

**Discussion:**

Inventories of medicinal plants confirm the persistence of traditional knowledge and reflect the need to recognize and respect this cosmovision. Many species are gathered in wild environments. The most important illnesses or diseases recorded in the present inventory are also mentioned in different studies, suggesting that they are common health problems in the rural communities of Mexico.

**Conclusions:**

Medicinal plants are essential for ethnic groups in Oaxaca. It is necessary to recognize and understand the complex ancestral processes involved in the human-nature interaction and the role of these processes in the conservation of biodiversity and in the survivorship of ethnic groups that have persisted for centuries. Finally, this study serves as a wake-up call to respect those worldviews.

**Supplementary Information:**

The online version contains supplementary material available at 10.1186/s13002-020-00431-y.

## Background

In terms of traditional medicinal knowledge, Mexico is recognized as the second most important country in the world, with a great ancestral tradition and richness in the use of medicinal plants, just after China. Inside the Mexican territory, which is characterized by a wide range of environmental conditions, approximately 4500 species [1, 2] are used by the 56 ethnic groups that occupy it [3]. The ancestral presence of these ethnic groups and their interactions with their surrounding environments have allowed the development of multiple local forms of knowledge and uses of natural resources to satisfy different needs [4].

Historical data and archaeological records indicate that for more than 5000 years, human groups in Mesoamerica have used numerous plants for magical, religious, and healing purposes. Although several of these plants are considered sacred and are associated with divinities, they also play important roles in disease-health processes [5–8]. Currently, most of these plants are still used for their same historical purposes and have been passed down through oral tradition among group members, mainly in rural areas [9, 10].

At the national level, Oaxaca is recognized as a state with deep traditional knowledge of medicinal plants among its 19 ethnic groups, including Mestizos and Afro-descendants [11]. This recognition has motivated the development of various studies on the traditional knowledge and use of medicinal plants within these groups. The research has included analyses of the roles of one or several species in the pharmacopeias of ethnic groups [7, 12], studies on complete local floras used in traditional medicine, and general ethnobotanical studies among the different ethnic groups (Chinantecos, Chocholtecos, Cuicatecos, Ixcatecos, Mazatecos, Mixes, Mixtecos, Nahuas, Triquis, Zapotecos, and Zoques) [4, 9, 10, 13–34].

Medicinal plants within traditional cultures are respected due to their effectiveness in treating various diseases and have shaped systems of local medical knowledge [13, 24], which in turn have been documented from different perspectives since ancient times, some valuing the empirical knowledge and others denigrating or even demonizing such knowledge and uses. However, in all available documentation, the existence of traditional doctors or healers and the accumulation of plants, animals, and even minerals used for the treatment of different ailments are recognized [35–39].

These complex traditional medical systems analyze the etiologies, symptoms, and developments of diseases themselves (e.g., [40, 41]). They also address the treatments that must be followed, which include the use of medicinal plants and series of cultural practices, such as *pedimento* ceremonies to the mountains (traditional practices such as praying and offering alcoholic beverages), in which healers follow other rituals to guarantee healing [12, 13, 24].

This topic has crossed the borders of local communities, and it has positioned itself in scientific research in several ways. Some studies have focused on the analysis of active compounds, demonstrating that the use of medicinal plants is based on rational criteria and that a relatively large proportion of plants produce the desired physiological effects [16, 37]. Other studies have demonstrated the use of plants combined with patent medicine to complement the “strengths” of plants or in cases of increased disease severity [42, 43]. Still others have analyzed the direct and collateral effects of medicinal plants on human bodily functions; these studies demonstrated the toxicity of various medicinal plants [2, 29, 42–44], meaning that Western science has focused on validating the use of medicinal plants outside of any cultural context, including the traditional practices and procedures that healers use and follow, which has placed many ancestrally used species in the sights of Western medicine [2, 45].

However, ethnobotanical research continues to yield data that substantially increase previous records on the various plant species used in traditional medicine. New data also provide evidence that medicinal plants that have been used for at least six centuries are still used at present, even when many other plants have been introduced from other places and been incorporated into traditional medical systems. As an example, when Spanish conquistadors brought smallpox, malaria, beriberi, and other diseases to America, native people probed their local remedies, along with those used by the conquistadors, to cure these unknown diseases [38]. Currently, new plants, illnesses, and remedies are reaching local communities, but traditional knowledge remains the mainstay of these medical systems.

In this context, the compilation of a present inventory of the medicinal plants used in the state of Oaxaca was carried out. Part of this inventory comes from an ethnofloristic exploration conducted between 2013 and 2016 in 84 Oaxacan municipalities located in three of the eight recognized priority terrestrial regions (PTRs) [46] (from now on the JF102 inventory). These regions include areas with high percentages of plant cover and high levels of ecological connection between ecosystems, and they are part of the important hydrological basins that feed these ecosystems [47]. These regions are also inhabited by rural communities, many of which belong to at least one indigenous ethnic group. It is important to remark that most of the ethnobotanical records from the JF102 inventory represent the first records made in the various visited municipalities and localities.

The results of the JF102 inventory indicated that the “medicinal” category was the most important use of plants, followed by “food.” This interesting pattern called our attention, along with the fact that during fieldtrips and walks, the guides told us about their experiences with medicinal plants, and in certain circumstances, we had the necessity to try the plants. Then, we considered it important to transmit this knowledge outside of the local communities, with the purpose of expanding the information and changing the concepts that Western people have regarding medicinal plants and traditional medical systems. As other authors have mentioned [32], traditional medicine remains very important in these rural communities, and traditional medicine is still available in moments of discomfort or need, even when people resort, to a greater or lesser extent, to Western medicine. Traditional medicine remains anchored to a series of practices, methods, and beliefs not only within a socioenvironmental context but also within each individual.

In the final stage of this JF102 inventory, we reviewed the medicinal plants that were reported in previous studies; this review led to the updating of records of medicinal plants in the state of Oaxaca, including records of different diseases and/or ailments that are treated with each medicinal plant; additionally, due to the pluricultural context of this biodiverse Mexican state, we described the contributions of traditional knowledge of medicinal plants among the different ethnic groups that inhabit the state of Oaxaca. This study informs the general proposed pattern regarding a strong link between some botanical families used for medicinal purposes; from this pattern, this study seeks to answer the following questions: Does the cluster of medicinal plants used in Oaxaca follow the same preferential patterns for some botanical families as those reported in publications? Is the probability of extracting plants of any vegetation type the same or are there groupings of vegetation types that are preferred? Is there a relationship between ethnic groups and the number of plants used for medicinal purposes or the kinds of illnesses/ailments the plants are used for?

## Materials and methods

### Updated traditional knowledge about medicinal plants in the state of Oaxaca

To update the traditional knowledge that exists about medicinal plants in the state of Oaxaca, a database was developed from two sources:

- One source, corresponding to an inventory, came from the results obtained during the ethnofloristic exploration of 84 Oaxacan rural municipalities between 2013 and 2016, located in three PTRs, named the JF102 inventory [46]. The choice of these regions was based on the richness of their ethnic composition, floristic diversity, and orographic complexity. This inventory was developed along with local people, who accepted the project and guided us to different places inside their territories where the plants used in the past and present could be found (for field methods, review [46, 48–50]). The local people also gave us information about the uses of the plants and told us about some personal experiences with them. Some of the voucher specimens were deposited in mexican herbaria like MEXU, FCIENCIAS, UAMIZ, and CIIDIR-Oaxaca. The deposition of the other portion is in progress.
  The database was developed with the following fields: botanical divisions, families, genera, species, classifications of medicinal use according to the World Health Organization [51], municipalities where the study was carried out, ethnic groups that inhabit those municipalities, vegetation types where the plants were collected, and the conservation risk statuses of the plants according to NOM-059-SEMARNAT-2010 [52] and the IUCN red list of threatened species [53].
- Another source corresponded to an exhaustive search of the scientific literature on medicinal plants in Oaxaca conducted in different electronic databases: Google Scholar, Commonwealth Agricultural Bureaux (CAB) Direct, Consorcio Nacional de Recursos de Información Científica y Tecnológica (CONRICYT), and Scopus. We reviewed all papers related to the knowledge of traditional medicine in the state of Oaxaca using the following keywords in Spanish and English: ethnobotany, traditional medicine, medicinal plants in the state of Oaxaca, and traditional uses of plants. Technical reports were also reviewed.

Once the database was complete, a review of the valid nomenclature of the species was carried out according to the International Plant Names Index [54]. Based on this information, descriptive analyses of different aspects were conducted, including the number of botanical families and species with medicinal uses, the most-represented botanical families, the vegetation types where the species came from, the origin or provenance of each species (native or introduced), and whether the species are listed in a risk category.

### Record of the different diseases and/or ailments that are treated with medicinal plants

The diseases and/or illnesses registered in the JF102 project and in the different publications were classified into 13 categories proposed by the World Health Organization (WHO) [51]: circulatory system (CS); digestive system (DS); endocrine, nutritional, and metabolic diseases (ENM); genitourinary system (GUS); injuries, poisoning, and certain other consequences of external causes (IP); infectious and parasitic diseases (IPD); mental and behavioral disorders (MBD); musculoskeletal system (MSS); nervous system (NS); pregnancy, childbirth, and puerperium (PCP); respiratory system (RS); skin and subcutaneous tissue disorders (SST); and neoplasms, carcinoma, and cancer (NCC). Additionally, the registered information was complemented with diseases of cultural-bound syndromes (CBS [32], giving a total of 14 categories.

### Record of the contributions of traditional knowledge of medicinal plants of the different ethnic groups that inhabit the state of Oaxaca

From the database, the number of medicinal species used among the studied Oaxacan ethnic groups was quantified. A multivariate analysis was applied to detect possible patterns in the distribution of ailments among ethnic groups based on the number of species used for each one. The total number of species used for each ailment by each ethnic group was considered. A hierarchical classification analysis (HCA) using Ward’s method on the Euclidean distance matrix was followed. Groupings obtained from the HCA were graphically evaluated with principal component analysis (PCA) on a correlation matrix (PAST 4.01 software).

## Results

### Traditional knowledge about medicinal plants in the state of Oaxaca

**Fig. 1:**
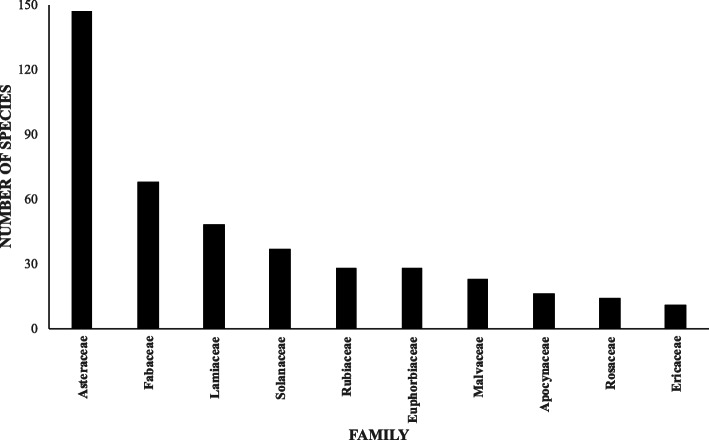
Ten most representative botanical families with medicinal plant species used in Oaxaca, Mexico

A total of 1032 species were fully identified as being used to treat different diseases and illnesses. Of these, 316 species were recorded in the JF102 inventory but were also reported in other studies, and of these, 138 species were contributions from the JF102 inventory, which corresponds to 13.37% of the total registered plants. Magnoliophytes had the largest record, with 942 species; there were 14 Coniferophytes and 64 Lycophytes and Polypodiophytes. In all, 164 families were registered, with the following being the families most used for medicinal purposes: Asteraceae, Fabaceae, Rubiaceae, and Malvaceae (Fig. 1). The remaining families comprised smaller numbers of utilized species. In addition to the 1032 species, 85 records were identified at the genus level, which were mentioned in several papers but were not taken into account in the results, as their identification at the species level is unknown.

In particular, the JF102 project provided records of 11 families not mentioned previously in the literature that are used to treat different diseases in the following systems of the human body, according to the WHO: Begoniaceae (DS, IP, IPD, PCP, SST), Calceolariaceae (IPD), Cannaceae (GUS), Costaceae (IP), Dioscoreaceae (CS), Ehretiaceae (SST), Heliconiaceae (SST), Martyniaceae (CS), Oxalidaceae (CBS), Polygonaceae (GUS), and Thelypteridaceae (DS) (Supplementary file 1: https://drive.google.com/file/d/1Km8EbtT3VTLrQcQdv1GLgaxy5Fzfcuel/view?usp=sharing).

**Fig. 2:**
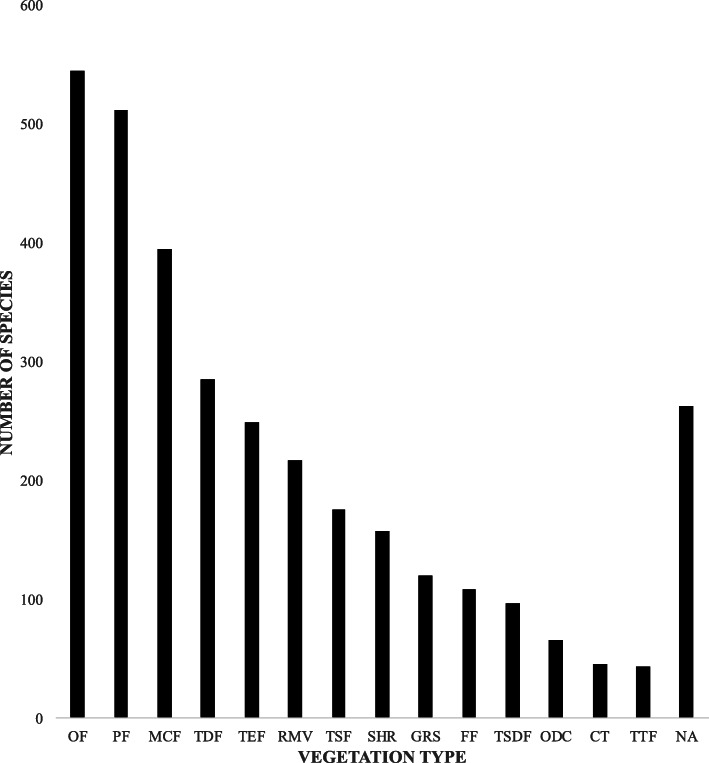
Number of medicinal plants recorded in each vegetation type in Oaxaca, Mexico. (Acronyms: OF oak forest, PF pine forest, MCF montane cloud forest, TDF tropical deciduous forest, NA not available, TEF tropical evergreen forest, RMV river-margin vegetation, TSF tropical semi-evergreen forest, SHR shrubland, GRS grassland, FF fir forest, TSDF tropical semi-deciduous forest, ODC oak-dominated chaparral, CT cardonal and tetechera, TTF tropical thorn forest)

Regarding the vegetation types among which the 1032 medicinal plants were obtained, 770 species were reported in 14 of the 26 vegetation types in Oaxaca (54%). The most important vegetation types were temperate forests, including oak forests (545), pine forests (513), and montane cloud forests (395; Fig. 2). Of these, 182 species were obtained from a single vegetation type, the most important being the oak forest, where 89 species were registered, some of them cataloged in a risk category (e.g., *Cyathea divergens* var. *tuerckheimii* (Maxon) R.M. Tryon, which is used for endocrine and nutritional diseases and is listed as “subject to special protection” [52] [NOM-059-SEMARNAT-2010] and *Quercus acutifolia* Née, which is used for the digestive system and is listed as a vulnerable species by the IUCN list [53]). The second most important vegetation type from which people obtain plants is the montane cloud forest, where 22 species were recorded, most of them by Rendón-Aguilar et al. [46] (e.g., *Bomarea edulis* [Tussac] Herb., used for childbirth and postpartum, and *Bartlettina tuerckheimii* [Klatt] R.M. King & H. Rob., which is used for musculoskeletal problems). None of the species collected in this vegetation type are registered in any risk category (Supplementary file 1). The remaining 262 medicinal plant species records did not include this information, and all of them were reported in the literature.

Of the 1032 recorded species, only 144 (13.95%) correspond to introduced species according to the “Plants of the World Online” portal from the Kew Science resource of the Royal Botanic Gardens.

**Fig. 3:**
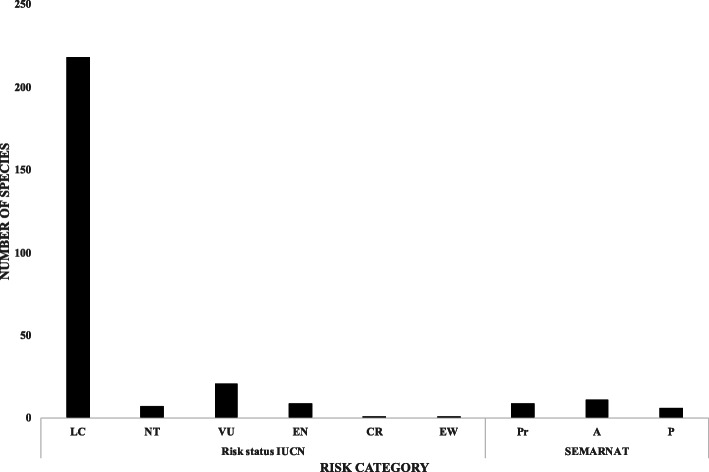
Number of species included in one of the risk categories by SEMARNAT [52] AND IUCN [53]

Regarding records of species that are considered in some risk category of NOM-059-SEMARNAT-2010 [52] or on the IUCN red list of threatened species [53], 272 species are listed, either by the IUCN (246), by SEMARNAT (15), or by both (11); *Liquidambar styraciflua* L., *Tecoma stans* (L.) Juss. ex Kunth, *Sambucus nigra* L., *Mimosa albida* Humb. & Bonpl. ex Willd., and *Matricaria chamomilla* L. are among the 16 species that are most widely used by at least four ethnic groups, but these species are listed by the IUCN as of least concern (Supplementary file 1 and Table 1, Fig. 3).

**Table 1:** Medicinal species used for more than six illnesses systems according to Pérez-Nicolás et al. [32] and WHO classification [51]

Plant species	Who illness system and	Ethnic groups	Risk status	Reference
*Calea urticifolia* (Mill.) DC.	CS, ENM, DS, NS, SST, IPD	Mes, Mix, Mxt, Tri		46(JF102), [4, 15, 16, 32, 55]
*Citrus aurantium* L.	DS, ENM, GUS, IPD, NS, PCP, RS, SST	Chi, Cho, Maz, Mix, Mes, Zap, Zoq		[13–15, 24, 25, 30–32, 42, 56]
*Equisetum myriochaetum* Schltdl. & Cham.	CBS, DS, ENM, GUS, MSS, IP	Cui, Maz, Mes, Mix, Nah, Zap, Zoq		46(JF102), [31, 32]
*Justicia spicigera* Schltdl.	CBS, CS, DS, ENM, GUS, NCC	Chi, Mes, Mxt, Tri, Zap, Zoq		[4, 13, 31, 34, 55]
*Lippia alba* (Mill.) N.E. Br. ex Britton & P. Wilson	CBS, DS, GUS, NS, PCP, RS	Chi, Maz, Mes, Mix, Mxt, Tri, Zap		[4, 14, 16, 24, 25, 32, 42, 55]
*Liquidambar styraciflua* L.	CBS, DS, IP, IPD, MSS, RS	Chi, Cui, Maz, Mes, Mix, Zap, Zoq	LC / -	46(JF102),[13, 31, 32, 57]
*Matricaria chamomilla* L.	DS, GUS, NS, PCP, RS, CBS	Chi, Cho, Mes, Zap	LC / -	[14, 20, 25, 32, 56, 58]
*Mimosa albida* Humb. & Bonpl. ex Willd.	CBS, MBD, NS, GUS, MSS, PCP	Chi, Cui, Maz, Mes, Mix, Mxt, Zap	LC / -	46(JF102), [14, 25, 27, 34, 55]
*Ocimum basilicum* L.	CBS, DS, GUS, MSS, NS, IPD	Mes, Miz, Mxt, Tri, Zap		[4, 15, 20, 25, 28, 32, 55]
*Ocimum campechianum* Mill.	CBS, ENM, IP, IPD, MSS, NCC, SST	Chi, Mes, Mix, Zap		46(JF102), [13, 15, 32, 55]
*Piper auritum* Kunth	CBS, DS, IP, GUS, IPD, SST	Chi, Cui, Maz, Mes, Mix, Mxt, Nah, Tri, Zap, Zoq		46(JF102) [4, 13–15, 20, 22, 30–32, 34, 55]
*Sambucus nigra* L.	CBS, IPD, MSS, NS, RS, SST	Cui, Maz, Mix, Nah	LC / -	46(JF102), [27]
*Solanum torvum* Sw.	DS, GUS, MSS, SST, CBS, NS	Mix, Zap, Zoq		[15, 20, 31, 55]
*Tecoma stans* (L.) Juss. ex Kunth	CBS, ENM, DS, SST, IPD, PCP	Cho, Ixc, Mes, Mix, Mxt, Zap, Zoq	LC / -	[15, 25, 31, 33, 55, 56, 59, 60]
*Tithonia diversifolia* (Hemsl.) A. Gray	DS, ENM, GUS, IP, MSS, IPD, SST	Chi, Mes, Mix, Mxt, Tri, Zap, Zoq		46(JF102), [4, 15, 20, 22, 30–32, 34, 55]
*Tournefortia hirsutissima* L.	DS, ENM, IPD, MSS, RS, SST	Zap		46(JF102), [34]

### Diseases and/or conditions that are treated with medicinal plants

**Fig. 4:**
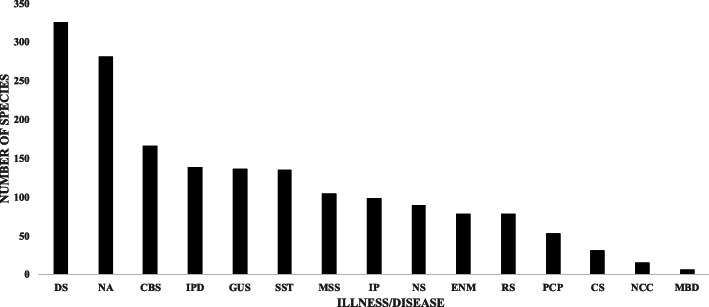
Number of medicinal plant species used for different illness system, according to Pérez-Nicolás et al. [32] and WHO classification [51]

Information from the literature and the JF102 inventory allowed us to register 14 diseases/illnesses [32, 51]; the ailments that registered the highest number of medicinal plants were as follows (Fig. 4).

- Conditions of the digestive system (DS), which included vomiting, diarrhea, colic, and stomach pain. For this system, 325 species were registered, belonging to almost 50% of the botanical families in the inventory. Some examples are *Costus pulverulentus* C. Presl and *Cirsium pinetorum* Greenm, which are used in infusions to cure dysentery and gastritis, respectively.
- Culture-bound syndromes (CBS), which included fright, evil eye, ritual cleansing (limpia), tiredness, courage, bad air, nightmares, and coldness in the body, among others; 166 species belonging to 64 botanical families were registered for these syndromes, the most important being Asteraceae, Fabaceae, and Lamiaceae. Some interesting examples include *Lepidaploa tortuosa* (L.) H. Rob., which is used to cure “snake scare” when a person sees a snake; *Clidemia setosa* (Triana) Gleason, which is used for the “infidelity scare” following the “doctrine of signatures” (in this case, the petiole of the leaf is shaped like a woman’s vagina); *Notholaenoid hemionitis* (Dev.) Christenh. and *Crotalaria sagittalis* L., which are used in infusions to stop children from urinating in their bed; and *Helicteres guazumifolia* Kunth, which are given to children to suck when the children are young and are slow to start talking.
- Problems of the genitourinary system (GUS), which include bleeding, prostate pain, and womb pain; 137 species included in 58 families were recorded to treat these ailments, mainly in the families Asteraceae and Lamiaceae. Several species are used for kidney ailments: *Borreria remota* (Lam.) Bacigalupo & E.L. Cabral, *Ruprechtia fusca* Fernald, and *Alsophila firma* (Baker) D.S. Conant (the latter is also used for prostate problems and diabetes). *Quercus polymorpha* Schltdl. & Cham. is used to cure infections in the urinary tract; in Spanish, this disease is called “mal de orín”.

**Fig. 5:**
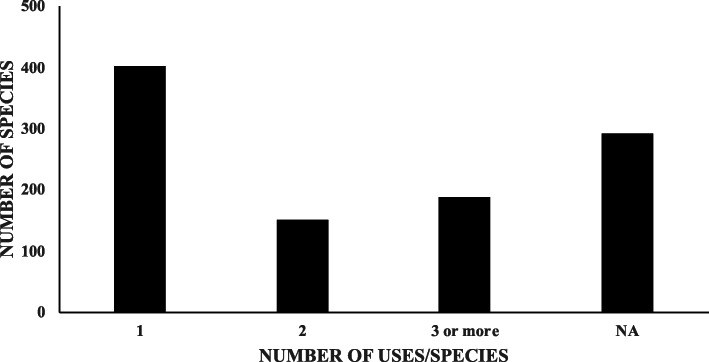
Number of medicinal plant species that attended one, two, three or more illness systems. The number of species with no records is indicated (NA)

Of the 1032 species records, 741 included a specific description of the medicinal use of the species. Of these, 54.25% had one use in one of the 13 categories, 20.2% were used for two systems and 25.5% were used for three or more systems (Fig. 1). The remainder of records (291) only mentioned that the corresponding species were medicinal plants (Fig. 5). Seventeen species are widely used to cure at least six different diseases or disorders related to different systems (Table 1); of these, the vast majority (12 species) are native species. The exceptions include two species of citrus (*Citrus limon* (L.) Osbeck, *Citrus aurantium* L.), a species of basil (*Ocimum basilicum* L.), elder (*Sambucus nigra*), and chamomile (*Matricaria chamomilla*), which are introduced species.

### Contributions of traditional knowledge of medicinal plants of the different ethnic groups that inhabit the state of Oaxaca

**Fig. 6:**
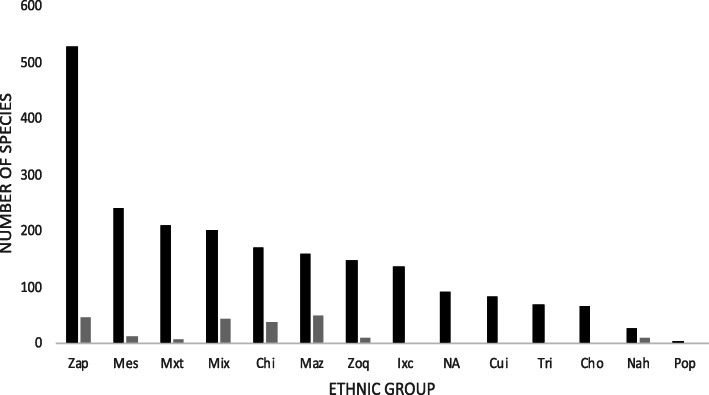
Number of medicinal plants recorded for each ethnic group in Oaxaca, Mexico (black bars=total number of species recorded; gray bars=species added by JF102 project)

Of the 19 ethnic groups that occupy the Oaxacan territory, including Mestizos and Afro-descendants [11], the consulted literature contains studies on medicinal plants or records of medicinal plants in twelve ethnic groups (63%; Fig. 6). In the case of the JF102 inventory, at least one species was linked to at least one of each of these twelve ethnic groups.

The ethnic groups with the greatest numbers of recorded medicinal plants were the Mestizo, Mixe, Mixtec, and Zapotec ethnic groups. In contrast, the ethnic groups with the lowest recorded numbers were the Nahua and Popoloc ethnic groups. The JF102 inventory comprised 38.46% of the records of medicinal plants from the Nahua ethnic group and between 20% and 30% of the records from the Chinantec, Mazatec, and Mixe ethnic groups (Fig. 6).

Regarding the medicinal plants used among the different ethnic groups within the Oaxacan territory, the 17 species used to cure the greatest number of diseases (Table 1) were also the most widely used species in Oaxaca in terms of the number of ethnic groups who use them, with the exception of *Solanum torvum* Sw., which was registered in three ethnic groups; *Matricaria chamomilla*, *Calea urticifolia* (Mill.) DC., *Sambucus nigra*, and *Ocimum campechianum* Mill., which were registered in only four ethnic groups; and *Tournefortia hirsutissima* L., which, despite being a species used to cure six diseases, was registered only among Zapotec communities.

**Fig. 7:**
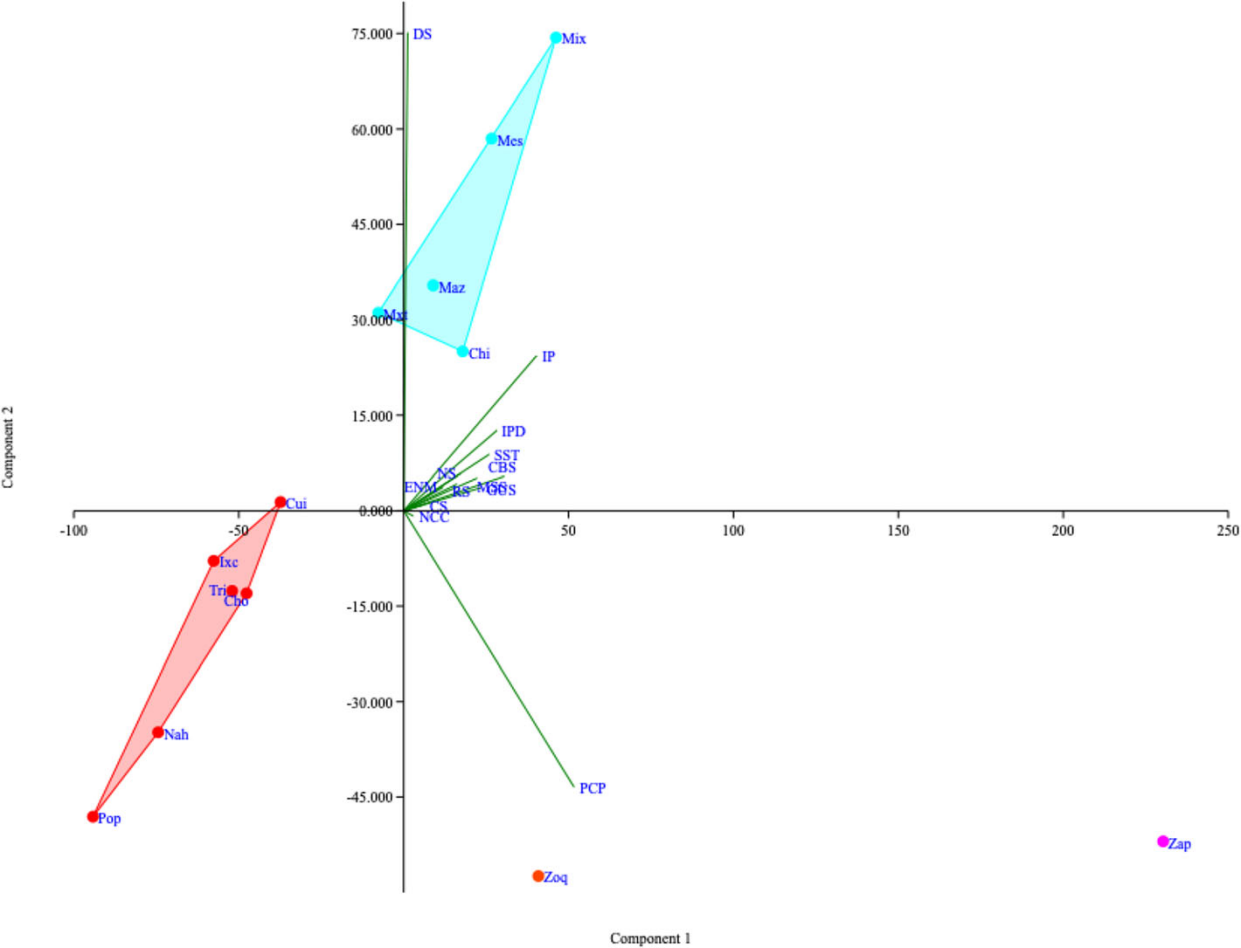
A hierarchical classification analysis (HCA) using Ward’s method on the Euclidean distance matrix was followed. Groupings obtained from the HCA were graphically evaluated with principal component analysis (PCA) on a correlation matrix (PAST 4.01).The first two axes of the PCA explained approximately 97% of the variance.

Multivariate analysis revealed groupings between ailments and ethnic groups (Fig. 7). The first two axes of the PCA explained approximately 97% of the variance. The first axis shows that PCP-, DS-, IP-, and IPD-related ailments were the ailments for which the largest number of species (more than 250 by each ethnic group) are used among the Zapotec, Zoque, Chinantec, Mazatec, Mestizo, Mixe, and Mixtec ethnic groups (right side of the PCA). The rest of the ethnic groups use fewer than 250 species among all ailment categories (left side of the PCA). The second axis separates the ethnic groups that use more medicinal plants for DS-, IP-, and IPD-related ailments (the Mixe, Mestizo, Mazatec, Mixtec, and Chinantec ethnic groups, upper side of the PCA) from those that use more medicinal plants for PCP-related ailments (the Zapotec and Zoque ethnic groups, lower side of the PCA).

## Discussion

Given the great biodiversity of the Mexican territory, as well as its complex physiography and geological history, it is to be expected that there are still various regions of the country that have been very scantly explored despite the enormous efforts of naturalists and researchers from the colonial epoch to the present. This lack of research is related to biodiversity in a broad sense but is also related to traditional knowledge and the use of natural resources. One of the uses of natural resources that has been of primary importance for the diverse cultures of the world and of Mexico, as well as for explorers and researchers, is that of medicinal plants. The purposes, objectives, and approaches of related research are varied, but they share the need to show that there is a large accumulation of plant species that are used to cure diseases, discomforts, and pestilences [1, 2, 35–40]. It has also been revealed that the use of medicinal plants involves a cosmovision that includes aspects of an individual’s personality, his/her history, and relationships with the rest of the community, as well as the existing link with the environment [38]. For these reasons, inventories of medicinal plants confirm the persistence of traditional cultures and their resistance to the great socioeconomic and political changes that occur around them and, ultimately, reflect the need for these ways of life to be recognized and respected in their own dimensions.

Mexico ranks second worldwide in the number of registered medicinal plants (4500) [61], and the present study shows that this value increases with each publication related to the topic; this trend also applies to Oaxaca. The most recent general inventory was published by Caballero et al. [62]. More recent reviews have been published corresponding to certain groups of medicinal plants, such as those used for antidiabetic purposes [59] or for gastrointestinal disorders [55]. The results of field collections carried out in the state of Oaxaca between 2013 and 2016 during the creation of the JF102 inventory [46] again demonstrate the existence of a large body of knowledge about traditional medicine that prevails in many indigenous and Mestizo communities. In this inventory, 139 species (13.6%) were added to the long list of medicinal plants reported in the present literature, and in addition to these species being new records, their relevance also corresponds to the places where they were collected. These botanical records are strongly linked to cultural manifestations, so the existence of concepts such as “viper scare,” “evil eye,” “lightning scare,” and “bad air,” which have been reported since colonial times, are still valid and current in the cosmovisions of their peoples.

### Traditional knowledge about medicinal plants in the state of Oaxaca

The most important botanical families found in the literature and in the JF102 inventory were Asteraceae, Fabaceae, Rubiaceae, and Malvaceae. This pattern agreed, in part, with that reported by Caballero et al. [62], who mentioned Asteraceae, Lamiaceae, Solanaceae, and Verbenaceae as the most important families selected for medicinal properties; this pattern also agrees with the results of Luna-José and Rendón-Aguilar [25], who reported Asteraceae, Fabaceae, and Solanaceae as the most important families; and with the results of Weckerle et al. [63], who stated that the most-used families among the Popoloc ethnic group were Asteraceae, Piperaceae, Acanthaceae, Amaranthaceae, and Lamiaceae. This selection of families could be related to certain active ingredients with therapeutic effects, as mentioned by Caballero et al. [62]. Various species of the Solanaceae family, among others, have been studied from a phytochemical basis, and certain active ingredients have been obtained, some of which corroborate the roles of these plants in traditional medicine while others contain compounds that may be threatening to human health [22].

Conversely, there are some botanical families that are poorly represented in this inventory, of which 11 were not recorded in previous studies (Begoniaceae, Calceolariaceae, Cannaceae, Costaceae, Dioscoreaceae, Ehretiaceae, Heliconiaceae, Martyniaceae, Oxalidaceae, Polygonaceae, and Thelypteridaceae). In some cases, such as for Dioscoreaceae, this result is strange because its medicinal properties are widely recognized in local communities but have not, for some reason, been included in previous studies. These families are rarely used since they were reported with only one genus and one species (with the exception of the Begoniaceae family), and each of these is applied to alleviate a disease of a single system [46]. In this context, 13% of species added by the JF102 inventory were members of these “new families” not mentioned in previous studies.

It is worth noting that in some of the reviewed papers, most of the plants with the highest uses are introduced [19, 43], highlighting the importance of managed and/or cultivated plants that have demonstrated efficacy as medicine and were, for this reason, widely disseminated among the indigenous peoples who cultivate them in their homes (e.g., orchards) to have them available. Such is the case for *Bougainvillea spectabilis* Willd. (bugambilia), *Marrubium vulgare* L. (marrubio), *Matricaria chamomilla* L. (manzanilla), *Mentha piperita* L. (mint), and *Ruta chalepensis* L. (ruda), among others. However, these reports also included native plants such as *Dysphania ambrosioides* (L.) Mosyakin & Clemants (epazote), and *Psidium guajava* L. (guava). Hernández-Ruíz et al. [58] reported the presence of a large quantity of medicinal plants, followed by a smaller quantity of edible plants, and many plants in both of these categories were introduced as follows: *Allium sativum* L. (garlic), *Bougainvillea spectabilis* (bugambilia), *Eucalyptus camaldulensis* Dehnh. (eucalyptus), and *Ricinus communis* L. (ricino oil), among others.

However, in the present study, most species with some medicinal use are native, which can be considered a qualitative indicator that the majority of plants reported in this study have been used since ancestral times and that the corresponding traditional knowledge has persisted to date.

Although several species, including the most widely used species, are listed in the least concern risk category, they are species with medium to high levels of abundance within Oaxacan communities (personal observation). Although many of the studied plants are gathered from wild populations, others are tolerated or even cultivated in backyard orchards or near homes [19, 43]. When medicinal plants are gathered exclusively from wild populations, people cut only the useful parts in small quantities and gather only when the plants are needed. Only in a few cases was full extraction reported. During different moments throughout the development of the JF102 inventory, we verified practices such as pruning, harvesting fruits, and extracting tree bark fragments; full extraction was not recorded.

### Diseases and/or conditions that are treated with medicinal plants

Data from the literature and the JF102 inventory showed that the greatest number of medicinal plants were used to cure or treat diseases and/or illnesses of the digestive system; this result agreed with the results of other studies that indicated the same pattern between the Chocholtec [56] and Zoque [30, 31] ethnic groups. In two papers, Heinrich et al. [16, 19] mentioned several genera that were widely used to treat diseases associated with the digestive system among the indigenous peoples of Oaxaca, such as *Annona*, *Artemisa*, *Carica*, *Citrus*, *Mentha*, *Persea*, *Psidium*, *Ruta*, and *Tagetes*; these species are consistent with the data reported in this inventory. In contrast, among the Mixtec ethnic group, Valdés-Cobos [28] recorded more plants used to cure diseases associated with the respiratory system, and the number of plants used for diseases of the digestive system ranked in second place. This result also indirectly indicates the health problems that these digestive system diseases represent in rural communities in Mexico such that traditional knowledge has focused significant efforts on their control or healing.

Medicinal plants used for culture-bound syndromes represent the third most important category in terms of the number of plants used by Oaxacan ethnic groups; this result reflects the persistence of the Mesoamerican cosmovision, despite fear, distrust, and even rejection during the colonial epoch [39] and still to the present. Since colonial times, different individuals, mainly foreign individuals, have contributed to the documentation of this traditional knowledge. In some publications, we found that many species (or, at least, some genera) had already been mentioned in early works written by scholars, physicians, and naturalists since the colonial epoch [35, 36]. An example corresponds to the genus *Bursera*, a group of several resin-producing species or *copal*. This term “ … became the European common name of one of the main groups of resins of American origin whose medical use consisted, on the one hand, in fumigations and incense sticks to “purify and correct the air” from the bad smell that galenism identified with its contamination, “infection” or “corruption”, on the other, to combat, with its quality or “hot temperament”, disorders of causes cold, such as headaches, colds and faints, and also in local applications, by ointments and plasters against all kinds of pain. They were also used for the preparation of varnishes and in Christian religious ceremonies, such as substitute for incense” [35]. Another example corresponds to different species of Euphorbiaceae (mainly *Croton* spp.), considered American plants that produce *sangre de drago* and were used as purgatives. In this inventory, several species were associated with the digestive system [35] (Supplemenatry file 1). Both species of *Nicotiana* (*N. tabacum* L. and *N. glauca* Graham), called *piciete* or *pixiote*, were referred to in the earliest records for their use in the curing of cultural ailments [36]. In all cases, the mentioned uses are still the same.

Even when a particular illness, pain, or disease can be clearly identified, its origin remains unclear. In some cases, these ailments can be associated with “envy,” “evil eye,” or personal problems. These conditions that can create health problems are not completely considered in Western medicine or in ethnobotanical studies. Thus, categorizing or classifying them only in terms of their symptoms without considering the origins can lead to simplistic classifications. For example, if a person exhibits weight loss, inappetence, and a haggardly constitution, Western medicine may conclude that he/she has anemia or parasites. A traditional healer will search deep within the patient, his/her personal problems and/or conflicts, trying to find the origin of the problem. The delineation between culturally bound syndromes and a digestive or blood problems is not clear. Any classification of an illness, care system, or medicinal plant is not sufficiently precise to include the range of situations associated with the disease, illness, or pain, or the reasons why some plants are used (62). This pattern is part of the breakdown of Western medicine and Western science, and reflection about the ways we look for health problems inside local communities and the remedies/treatments that traditional healers follow is necessary to develop new strategies and methodologies to document different aspects of traditional knowledge of medicinal plants and traditional medicinal systems.

### Contributions of traditional knowledge of medicinal plants of the different ethnic groups that inhabit the state of Oaxaca

The inventory indicated that the Mestizo, Mixe, Mixtec, and Zapotec ethnic groups used a large proportion of medicinal plants. This pattern was largely a function of the number of municipalities studied. Of the 28 sources consulted, 28.6% corresponded to studies carried out in Zapotec communities, 17.9% in Mixe communities, and only 14.3% in Mixtec communities. Likewise, the highest number of records documented in the JF102 inventory [46] corresponded to the Zapotec (33%), Mixe (19%), Mazatec (18%), and Chinantec (13%) ethnic groups. However, this pattern was only observed for the Mixtec and Zapotec people but not for the Mixe people, whose population is less than that of the Mazatec people [60]. It should also be noted that most of these studies aimed to register useful plants in general; therefore, the systematic monitoring of medicinal uses was not addressed in depth (e.g., [23, 25, 64–66]) and could represent another factor influencing this pattern.

Despite these factors that could bias the numbers obtained among ethnic groups, some patterns could be detected from the multivariate analysis, suggesting a differential value of some ailments among the ethnic groups with more medicinal plants records. For example, the Zapotec and Zoque ethnic groups assigned more medicinal plants to PCP-related ailments, while Chinantec, Mazatec, Mestizo, Mixe, and Mixtec assigned more plants to DC-, IP-, and CBS-related ailments. This pattern does not depend on the extension of the territory or the number of inhabitants. However, some ethnic groups had few records in all categories, which made it difficult to associate the records with any of the ailments.

The highlighting of these results was conducted to demonstrate the need to continue building inventories and expanding research in different ethnic groups. The JF102 inventory of useful ethnoflora from Oaxaca [46] added 139 species of medicinal plants to the record, and the contribution breakdown by ethnic group was 21% for the Mixe ethnic group, 22% for the Chinantec ethnic group, 30% for the Mazatec ethnic group, and 38% for the Nahua ethnic group. In contrast, there was little contribution associated with the Zoque people (6.8%) due to the recent papers of Geck et al. [30, 31]; with the Mixtec people (3.4%), thanks to recent works by different researchers [4, 28, 60, 67]; or with the Zapotec people (8.9%), which is the most historically studied ethnic group [19, 20, 22, 25, 32, 34, 57, 68].

Nevertheless, the contributions of the JF102 inventory were reflected not only in terms of the reported number of families, ailment categories, and ethnic groups. The contributions also represent a great human effort to enter these territories and gain the trust of the people. Thus, from 1971, with the first publication by Lipp [13] with Chinantecos, to the most recent regarding the traditional knowledge of Zapotecos from the Central Valleys [34], only 34 municipalities had been studied. If we disregard the previously studied municipalities, the JF102 inventory added ethnobotanical records of medicinal plants from 75 municipalities. In terms of ethnic groups, only one ethnic group was added. However, records of medicinal plants inside each ethnic group help reinforce and enrich records of traditional knowledge.

## Conclusions

The traditional uses of medicinal plants are essential for the people of Oaxaca. Twelve ethnic groups (63%) maintain the historical memory of medicinal plants and, in their everyday practices, maintain the knowledge and use of these species. These plants are both functional and effective in the local context in which they are applied and/or consumed, given the dose, frequency of use, and physiological state of the individual. Many of these plants are gathered from the wild, mainly from oak, pine, and montane cloud forests, which are considered areas of species diversification and of the highest plant cover in Mexico. This reinforces the thesis of a close relationship between ethnic diversity and high levels of biodiversity proposed by different authors [25, 69, 70]. The traditional knowledge of the rest of the ethnic groups regarding medicinal plants remains unknown, at least in terms of papers, theses, or books, and more research is necessary to continue updating this inventory. From the present study, it is clear that studies such as this try not to validate traditional knowledge but to make it valid to Western science.

To the extent that we recognize and understand these interactions, we will allow the complex ancestral processes involved in human-nature interactions to continue and, with them, the conservation of biodiversity. This reflection comes from the widely recognized correlation between the presence of ethnic groups and more preserved geographic areas. Given the increasingly strong evidence that Western socioeconomic systems, as well as acculturation, have led to the fragmentation and breakdown of nature, it is necessary to understand that there are many ways of seeing and living in the world. These perspectives are functional to traditional communities, which are why they have persisted despite the conquests, plagues, and exploitation of natural resources by the great oligopolies and monopolies; it is also why these studies are a wake-up call to respect those worldviews. Ethnobotanical studies are conducted in the context of the traditional use and knowledge of biodiversity, which is a world heritage that is protected by local ethnic groups. This inventory is an effort to contribute to this vital heritage.

## Supplementary Information

**Supplementary file 1.**

## Data Availability

This information corresponds to Supplementary file 1. The datasets used and/or analyzed during the current study are available from the corresponding author on reasonable request: https://drive.google.com/file/d/1Km8EbtT3VTLrQcQdv1GLgaxy5Fzfcuel/view?usp=sharing.

## Abbreviations

PTR: Priority terrestrial region
MEXU: Instituto de Biología, Universidad Nacional Autónoma de México
FCIENCIAS: Facultad de Ciencias, Universidad Nacional Autónoma de México
UAMIZ: Universidad Autónoma Metropolitana, unidad Iztapalapa
CIIDIR-Oaxaca: Centro Interdisciplinario de Investigación para el Desarrollo Integral Regional, unidad Oaxaca
NOM-059-SEMARNAT-2010: Norma Oficial Mexicana 059-Secretaría de Medio Ambiente y Recusos Naturales-2010
CAB Direct: Commonwealth Agricultural Bureaux
CONRICYT: Consorcio Nacional de Recursos de Información Científica y Tecnológica
WHO: World Health Organization
CS: Circulatory system
DS: Digestive system
ENM: Endocrine, nutritional, and metabolic diseases
GUS: Genitourinary system
IP: Injuries, poisoning, and certain other consequences of external causes
IPD: Infectious and parasitic diseases
IUCN: International union for conservation of nature
MBD: Mental and behavioral disorders
MSS: Musculoskeletal system
NS: Nervous system
PCP: Pregnancy, childbirth, and puerperium
RS: Respiratory system
SST: Skin and subcutaneous tissue disorders
NCC: Neoplasms, carcinoma, and cancer
CBS: Cultural bound system
HCA: Hierarchical classification analysis
PCA: Principal component analysis
